# Using Attribution Sequence Alignment to Interpret Deep Learning Models for miRNA Binding Site Prediction

**DOI:** 10.3390/biology12030369

**Published:** 2023-02-26

**Authors:** Katarína Grešová, Ondřej Vaculík, Panagiotis Alexiou

**Affiliations:** 1 Central European Institute of Technology (CEITEC), Masaryk University, 625 00 Brno, Czech Republic; 2 Faculty of Science, National Centre for Biomolecular Research, Masaryk University, 625 00 Brno, Czech Republic

**Keywords:** miRNA target prediction, CLASH, deep learning, interpretation, visualization

## Abstract

**Simple Summary:**

MicroRNAs are small non-coding RNAs that play a central role in many molecular processes, but the exact rules of their activity are not known. In recent years, deep learning computational methods have revolutionized many fields, including the microRNA field. While making accurate predictions is important in biomedical tasks, it is equally important to understand why models make their predictions. Here, we present a novel interpretation technique for deep learning models that produces human readable visual representation of the knowledge learned by the model. This representation is useful for understanding the model’s decisions and can be used as a proxy for the further interpretation of biological concepts learned by the deep learning model. Importantly, the presented method is not tied to the model or biological domain and can be easily extended.

**Abstract:**

MicroRNAs (miRNAs) are small non-coding RNAs that play a central role in the post-transcriptional regulation of biological processes. miRNAs regulate transcripts through direct binding involving the Argonaute protein family. The exact rules of binding are not known, and several in silico miRNA target prediction methods have been developed to date. Deep learning has recently revolutionized miRNA target prediction. However, the higher predictive power comes with a decreased ability to interpret increasingly complex models. Here, we present a novel interpretation technique, called attribution sequence alignment, for miRNA target site prediction models that can interpret such deep learning models on a two-dimensional representation of miRNA and putative target sequence. Our method produces a human readable visual representation of miRNA:target interactions and can be used as a proxy for the further interpretation of biological concepts learned by the neural network. We demonstrate applications of this method in the clustering of experimental data into binding classes, as well as using the method to narrow down predicted miRNA binding sites on long transcript sequences. Importantly, the presented method works with any neural network model trained on a two-dimensional representation of interactions and can be easily extended to further domains such as protein–protein interactions.

## 1. Introduction

MicroRNAs (miRNAs), first discovered in Caenorhabditis elegans in 1993 [1,2], are an abundant class of small (~17–25 nt long) non-coding RNAs that regulate gene expression at the post-transcriptional level [3,4,5,6]. Mature miRNAs are loaded into the Argonaute (AGO) protein, and along with other proteins, form the miRNA-induced silencing complex (miRISC). miRNAs guide the miRISC, through partial base pairing, to target messenger RNAs (mRNAs) [7,8]. Such targeting may lead to translational repression and deadenylation-induced mRNA degradation [9,10]. Several studies have revealed miRNAs involvement in not only normal physiological processes but also pathologies [11,12]. The abnormal expression or function of miRNAs has been closely related to diverse human diseases, such as cancers. miRNAs are thus emerging as novel endogenous bio-targets for diagnostics and therapeutic treatments [13,14]. Understanding miRNA-involved cellular processes, including a clear picture of regulatory networks of intracellular miRNAs, is, therefore, essential and critical for miRNA-targeted biomedicine [15,16]. The 5′ end of the miRNA, and especially the hexamer spanning nucleotides 2–8, were very early identified as important for miRNA target recognition and termed the “seed” region [17]. Target recognition is primarily achieved via base pairing that involves the seed region [18]; however, seed pairing is not always sufficient for functional target interactions, and additional interactions with the miRNA 3′ end may be necessary for specific targeting [19]. Several experimental methods for identifying miRNA:target site pairs interactions have been developed, discovering abundant classes of non-seed interactions [20,21,22].

Experimental validation of functional miRNA:target pairs is a laborious process and computational tools can be utilized to simplify it. The first programs for the computational prediction of miRNA targets started to appear in 2003, shortly after it was suggested that miRNAs are widespread and abundant in cells [4,5,6]. Each mRNA can contain dozens of potential miRNA binding sites [23] and target prediction programs identify these binding sites and combine them into the final prediction on the level of the whole gene. Two main approaches for binding site identification are the “cofold” and the “seed” heuristics [24]. The “cofold” heuristic computes the hybridization energy of miRNA and the binding site sequences [25,26,27]. It also produces a base pairing pattern of two input sequences, providing a way to visualize the miRNA:binding site interaction. However, this computation does not take into account the AGO protein affecting the interaction, resulting in poor predictive power [28]. The “seed” heuristic uses the relaxed seed region to scan the target for potential binding sites. This approach outperforms the “cofold” heuristic [28], but it misses non-seed interactions, amplifying the seed bias. It also lacks the base pairing visualization feature. Advances in experimental identification of miRNA binding sites [20,29] have enabled the rise of computational methods based on machine learning (ML) and especially deep learning (DL). DL methods are currently state-of-the-art in the field and are highly appropriate for uncovering the miRNA binding rules, where clear rules or features are unknown since they work with the raw data and compute the features themselves [28,30].

Despite the high accuracy of DL models, these models have several disadvantages that hinder their usability and interpretability. DL models trained for miRNA target site prediction often work with the fixed input length, giving the prediction score for the whole input sequence, even though it is known that miRNAs are only approximately 17–25nt long, and their target sites potentially even shorter. DL models are also infamous for being unable to directly interpret what they learn from the data. While making accurate predictions is important in biomedical tasks, it is equally important to understand the reason why models make their predictions. Although DL models are not designed to highlight interpretable relationships in data or to guide the formulation of mechanistic hypotheses, they can, nevertheless, be interrogated for these purposes a posteriori [31]. The lack of direct interpretability of DL models may keep them back from being widely used in the context of miRNA target site prediction, compared to less powerful but easily interpretable models, such as the seed measure.

In complex models, it is imperative to inspect parameters indirectly by probing the input–output relationships for each predicted example. Attribution scores, also called feature importance scores, relevance scores, or contribution scores, can be used for this purpose. They highlight the parts of a given input that are most influential for the model prediction and thereby help to explain why such a prediction was made. Techniques for obtaining the attribution scores can be divided into two main groups on the basis of whether they are computed using input perturbations or using backpropagation. Perturbation-based approaches [32,33,34] systematically change the input features and observe the difference in the output. For DNA sequence-based models, the induced perturbation can be, for example, a single-nucleotide substitution [33,35,36,37,38] or the insertion of a regulatory motif [39,40]. Backpropagation-based approaches [41,42,43,44,45,46,47] propagate an important signal from an output neuron backward through the layers to the input in one pass. This makes them more efficient than perturbation methods. While DL models are only as good as the data they were trained on, the interpretation technique is constrained by the used representation of data. The field of miRNA targeting is generally not interested in the specific sequence, but rather in the interactions between two sequences, namely the miRNA and target RNA. For the interpretation technique to point to the important interaction, this information has to be encoded in the data.

In this paper, we propose a novel interpretation technique for the miRNA target prediction models working with the 2D-binding representation of input sequences. The 2D-binding representation encodes interactions between sequences, allowing the interpretation technique to work in the context of interactions, not sequences. This interpretation provides an understandable visualization of the miRNA:target site interaction in the form of base pairing with the importance scores for each position. It can be further used as a proxy for studying the biological concepts learned by the neural network. We present several applications, such as identifying classes of miRNA binding activities (including seed and non-seed binding) and enhancing the target site predictions by narrowing it to the length of miRNA. All the code and data are available at https://github.com/katarinagresova/DeepExperiment.

## 2. Materials and Methods

### 2.1. Datasets and Models

MiRNA:target site interaction datasets introduced in Klimentova et al., 2022 [28] were retrieved from the GitHub repository (https://github.com/ML-Bioinfo-CEITEC/miRBind, date accessed: 9 December 2022). Positive miRNA:target interactions originates from the Helwak et al., 2013 CLASH experiment [29]. Klimentova et al., 2022 standardized the length of miRNA sequences to 20 nt, anchored by the 5′ end of the miRNA. The length of target sequences was standardized to 50 nt by centering and either clipping the sequence or extending it using the hyb reference [48]. These processed miRNA:target pairs were called the positive dataset. As explained in Klimentova et al., 2022, the negative set was constructed by matching real target sequences with random miRNAs from the same experiment excluding the miRNA:target pairs from the positive set.

**Figure 1:**
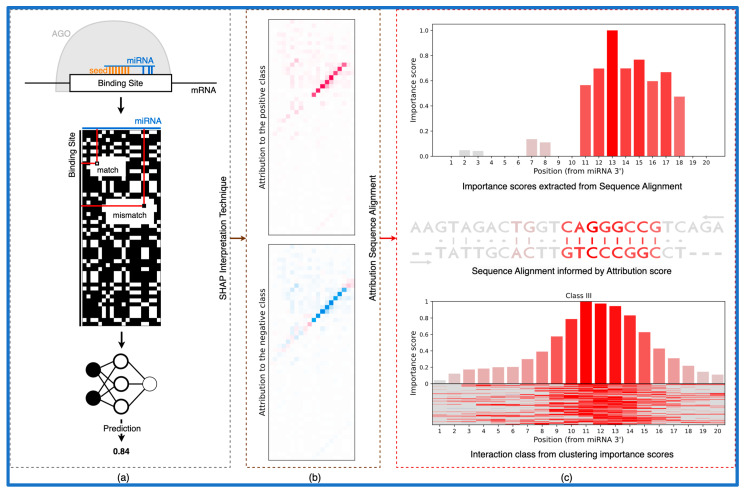
From the classical neural network to the biologically relevant representation. (**a**) Outline of a DL model workflow. (**b**) Interpretation method produces attribution scores for each pixel in the input. (**c**) Using the attribution scores to compute the interaction between sequences in the form of sequence alignment. In addition, we can compute the importance of each position for the interaction and use clustering to obtain interaction classes.

The trained models introduced in Klimentova et al., 2022 [28], namely *miRBind* and *CNN_model_1_20_optimized*, were downloaded from the GitHub repository (https://github.com/ML-Bioinfo-CEITEC/miRBind, Date accessed: 9 December 2022). Authors used a modified version of ResNet [49] as a miRBind architecture and a convolutional neural network architecture [50] for the CNN_model_1_20_optimized model. Both models use a two-dimensional representation of miRNA and the putative target site, in which any Watson–Crick binding nucleotide pair is represented by 1, and any non-binding pair by 0, as an input. For the miRNA of length 20 nt and target site of length 50 nt, the result is a 50 × 20 two-dimensional matrix of 1 s and 0 s (Figure 1a).

### 2.2. Attribution Scores

Attribution scores were computed using two implementations of the SHAP explanation method [47]—DeepExplainer and GradientExplainer—both available in the shap python package (https://github.com/slundberg/shap, date accessed: 9 December 2022). DeepExplainer implementation builds on a connection with DeepLIFT [45], while GradientExplainer builds on ideas from Integrated Gradients [44] and SmoothGrad [51]. Attribution scores highlight areas within the input that contribute positively or negatively to a model’s decision.

The SHAP explanation method is based on principles of the Shapley value. The Shapley value [52] is a widely used method for explaining the outputs of a model and understanding the relationship between the features of the data and the model’s predictions. By assuming that each feature is a “player” in a game where the prediction is the “payout”, the Shapley value provides a fair way to distribute the payout among the features. In this paper, we utilized the SHAP explanation method [47] that computes Shapley values with one innovation: the Shapley value explanation is represented as an additive feature attribution method, a linear model.

The SHAP explanation method requires a model, the data sample, and a set of background samples as input parameters. In this study, we selected 100 background samples to be optimal in terms of computational time and variation in importance scores (Figure S1). The variation in importance scores was used to measure the variation in results between models using different random background samples. We computed variation and computation time for numbers of background samples of 10, 50 and then adding 50 background samples up to 500. Each point was averaged over 10 runs.

The output of the SHAP method is a matrix with the same shape as the input data sample. In this study, we used samples in the format of a 50 × 20 2D matrix of 1s and 0s (as proposed by Klimentová et al., 2022 [28], Figure 1a); therefore, the output is a 50 × 20 matrix of SHAP values for each pixel in the input sample (Figure 1b). For each class, the input pixel with assigned positive SHAP value increases the model’s probability to classify the input as a given class and the negative value decreases the probability. As for the case of data from Klimentová et al., 2022 with only two classes, attribution scores for the positive and negative class differ only in the sign, pixel, with a high positive value for one class and a highly negative value for the other class.

### 2.3. Attribution Sequence Alignment

The attribution scores obtained from the SHAP explanation method produce the map of areas in the input that are positively and negatively contributing to the model’s decision. However, it is hard to see some biological features in this representation. Attribution sequence alignment is based in the principles of dynamic programming for the semi-global sequence alignment computed on top of attribution scores, transforming the attribution scores into the human readable visual representation of miRNA:target interactions.

The computation of attribution sequence alignment is based on two steps: (1) forward pass, where the dynamic programming matrix is filled (Algorithm 1), and (2) backward pass, where sequence alignment is computed by finding the highest-scoring path in the dynamic programming matrix. Parameters for the forward pass are the scoring matrix and opening and elongation penalty. The attribution scores for a given input (computed using the method described in Section 2.2.) are used as a scoring matrix. The opening and elongation penalty score is computed for each alignment separately, based on the values in the scoring matrix. The opening penalty is set to the 99th percentile score and elongation penalty to the 90th percentile score. This setting is highly incentivizing mismatches over insertions or deletions and longer bulges over shorter ones. The backward pass is computed the same way as in the original algorithm by Smith and Waterman [53].
**Algorithm 1.** Algorithm for computing the dynamic programming matrix for modified semi-global sequence alignment.***Input:****gene and miRNA sequences of length M and N, respectively; scoring matrix of shape MxN; opening and elongation penalty score.* ***Output:****Dynamic programming matrix DP.**1.* ***Initialization:****2.* *reverse the order of gene and miRNA to match the scoring matrix**3.* *remove negative scores from the score matrix**4.* *swap sign of scores for the mismatch positions in the scoring matrix**5.* *add the first row and column of zeros to the scoring matrix**6.* *initialize the first row and column of the DP matrix with zeros**7.* ***Dynamic programming:****8.* ***if****last row or column **then****9.* *penalty = 0**10.* ***else if****is opening gap **then****11.* *penalty = opening_penalty**12.* ***else****13.* *penalty = elonging_penalty**14.* ***end if****15.* ***for****i: 1 to M **do****16.* ***for****j: 1 to N **do****17.* *DP_i,j_ = max(DP_i,j−1_ − penalty,*     *DP_i−1,j−1_ + score_matrix_i,j_,*     *P_i−1,j_ − penalty)**18.* ***end for****19.* ***end for***

The outputs of the attribution sequence alignment algorithm are three sequences with the same length: (1) aligned miRNA sequence, (2) aligned binding site sequence, and (3) sequence of attribution scores for each position in the alignment. The first two sequences are obtained from the backward pass of the dynamic programming matrix and are describing the interaction base by base. The third part of the output is obtained from the interpretation matrix and describes the importance of each position for the interaction. For each aligned base pair, the corresponding score is taken from the interpretation matrix, and for the “deletion” or “insertion”, the score is set to zero. These outputs can be used to produce a biologically relevant representation of the interaction between the miRNA and the binding site, as captured by the model.

### 2.4. Importance Scores for miR-7 and miR-278 Binding

In vivo experimental mutagenesis data were extracted from Figure 1 from Brennecke et al., 2005 [54]. There are two mRNA:target site pairs with the length of 23 and 22 nt, respectively. We used the first 20 nt of miRNA sequences (starting from the 5′) and the whole target site sequences. Relative reporter activity values for mismatched positions were manually extracted from Figure 1c from Brennecke et al., 2005 and are shown in Supplementary Table S1 These data contain values for positions 1 to 10 and one aggregated value for the 3′ end.

Importance scores for *miR-7* and *miR-278* binding sites were computed using the miRBind model, the Deep SHAP interpretation method with 100 background samples, and attribution sequence alignment. We computed importance scores in 10 runs with different background samples, demonstrating the variability of the output. The importance scores starting from position 11 were averaged into one importance score representing the aggregated value for the 3′ end.

In silico mutagenesis (ISM) is a common interpretation technique from the group of perturbation-based approaches [35,36,39,55,56,57]. ISM is an alternate feature attribution approach that involves making systematic mutations to characters in an input sequence and computing the change in the model’s output due to each mutation. It is the computational analog of saturation mutagenesis experiments [58] that are commonly used to estimate the functional importance of each character in a sequence of interest based on its effect size of mutations at each position on some functional read-out, making it a good candidate for obtaining position importance scores for *miR-7* and *miR-278* binding sites. We conducted two versions of the ISM interpretation, termed here ISM Full and ISM Brennecke. In ISM Full, we systematically mutated each nucleotide in the input miRNA, changing it to three other possible nucleotides, and observed the model’s output. We also computed the model’s prediction for the original miRNA sequence and used it as a base value from which we subtracted the average of the model’s outputs for mutated inputs, resulting in an importance score for a given position. In ISM Brennecke, we performed only the mutations as described in the Figure 1 from Brennecke et al., 2005 and we used changes in the model’s outputs as importance scores.

### 2.5. Narrowing Peaks

Artificial data with planted seeds were constructed by inserting a seed sequence into a background gene. A background gene was created by generating a random RNA sequence in which all four bases occurred with equal probability. The first miRNA from the Klimentova et al., 2022 evaluation dataset was selected and the 10 nt seed region starting at the second position was extracted. We calculated the reverse complement of the extracted seed sequence and planted it into specified positions in the gene to create this artificial data. Artificial data with stitched binding sites were constructed from the binding site from the Klimentova et al., 2022 evaluation dataset. We selected the most abundant miRNA sequence and its positive and negative target sequences. The artificial target gene sequence was obtained by combining the positive and negative binding site of a given miRNA.

To obtain the model’s output peaks, we used the miRBind model to scan the gene sequence using a 50 nt window with a step size of 1 nt. For each position, we transformed the 50 nt gene window sequence and the miRNA sequence into a 2D-binding matrix and fed it through the miRBind model. The obtained score was added to the overall score for all positions in the current window. After computation, the overall score was normalized in each position by the number of output scores that were added to that position.

To obtain peaks using the interpretation of the miRBind model, we scanned the gene sequence in the same manner as in the previous method. For each position, we computed the model’s output score and, if the score was higher than 0.5, we interpreted the model at that position using DeepExplainer, obtaining an attribution matrix with a size of 50 times length of miRNA. Each position in the attribution matrix was scaled by the model’s output and added to the corresponding position in the overall attribution matrix. The overall attribution matrix had a size of the length of the gene times the length of the miRNA. To identify peaks from this matrix, for each position in the gene, we took the maximum value in the corresponding column.

To compute the alignment of miRNA with its binding site, we first smoothed the maximum score obtained from the overall attribution matrix and identified the local maxima. The window of size 50 nt around the local maxima was extracted from the gene sequence and the overall attribution matrix. The attribution sequence alignment method was used to compute the alignment and per-nucleotide importance scores in the selected window.

### 2.6. Comparing Models and Interpretation Methods

To compare position importance scores computed with our attribution sequence alignment method on attribution scores produced with a different interpretation method on different models, we utilized two models published by Klimentová et al., 2022—miRBind and CNN_model_1_20_optimized—and two interpretation methods available in the shap package—DeepExplainer and GradientExplainer. We computed position importance scores for the following pairs: miRBind model and DeepExplainer interpretation method, miRBind model and GradientExplainer method and CNN model and GradientExplainer interpretation method. The pair with the CNN model and DeepExplainer interpretation method was omitted due to the implementation problem of DeepExplainer method in the shap package.

We used 300 true positive samples from the evaluation set published by Klimentová et al., 2022 as our input data. We computed three sets of position importance scores using our attribution sequence alignment method on attribution scores produced with a different interpretation method on different models. To compare the results, we computed the Pearson correlation coefficient on the pairs of position importance scores. We also randomly shuffled a set of position importance scores in each pair and computed the Pearson correlation coefficient on these new pair. The P-value was calculated using the Wilcoxon rank sum test on correlation coefficients from original and shuffled samples.

## 3. Results

### 3.1. Using Attribution Scores to Interpret DL Models of miRNA:target Prediction

The main aim of the presented method is the interpretation of DL models which work on 2D base pairing representations of miRNA:target site interactions (Figure 1a). Previously, we have shown that such models outperform traditional “seed” or “cofold” approaches [28]. Given as the input to such a trained model on 2D miRNA:target data, we use DeepExplainer [47] to calculate attribution scores for each potential interaction on the 2D matrix (Figure 1b). We use principles of dynamic programming to calculate an optimal path through the binding and attribution matrices, which is in turn used to align the two sequences in a way informed by the attribution scores (Figure 1c). This alignment is interpreting what the trained model has learned, which takes into account several factors such as the interaction between the miRNA, the target site, and the AGO protein. Traditional “cofold” methods lack this information, and although they can produce a similar alignment, their predictive value is lower than that of the DL models [28]. In turn, this attribution sequence alignment is used to cluster putative binding sites into categories based on their predicted mode of binding (Figure 1c).

### 3.2. Attribution Scores Closely Correlate to In Vivo Experimental Data

**Figure 2:**
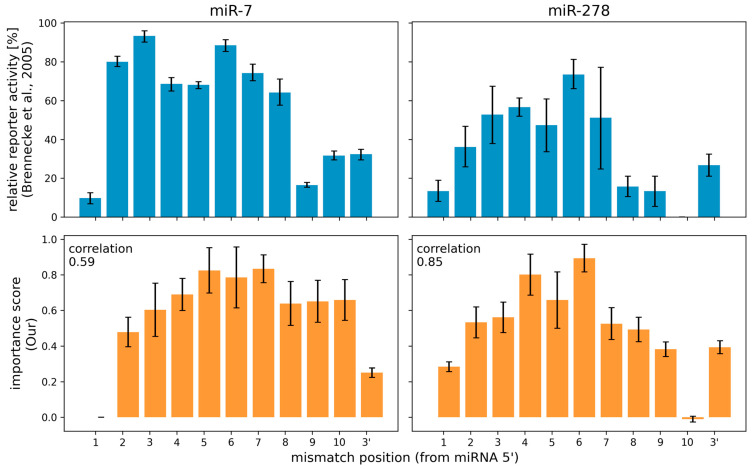
Comparison of relative reporter activity and importance score of *Drosophila*’s *miR-7* and *miR-278*. Values from Brennecke et al., 2005 comes from in vivo mutagenesis experiments. Our values are computed by the attribution sequence alignment method from an interpretation of the miRBind model trained on Helwak et al., 2013 Human Ago1 CLASH data. The correlation coefficient between relative reporter activity and importance score was computed using the Pearson correlation coefficient.

The interpretation method proposed here can be used to produce per-nucleotide importance scores to miRNA sequences within a miRNA:target site interaction. Brennecke et al., 2005 [54] performed an in vivo experiment, in which they systematically introduced single-nucleotide changes in a miRNA target site in order to produce mismatches at different positions of the miRNA:target site duplex. They then observed changes in the repression of the target gene for two miRNA:target site pairs in *Drosophila* (Figure 2). They reported that mutating specific single nucleotides conferred strong reduction in the ability of the miRNA to regulate its target. For *mir-7,* positions 2 to 8 were identified as most important, and for *miR-278,* positions 2–7 from the miRNA 5′ end.

We used as the input the miRBind model, which has been trained on Human AGO1 CLASH data, and we implemented three different interpretation methods (a) our attribution sequence alignment, (b) ISM Brennecke and (c) ISM Full (see Methods for details). We computed the importance of each position on the miRNA for the same two miRNA:target pairs as in Brennecke et al., 2005. Importance scores from our attribution sequence alignment were largely consistent with Brennecke et al.’s in vivo assay results (Figure 2). Notably, we see that the diminished importance of nucleotide 1 and the 3′ end are correctly interpreted using our method, corresponding to the experimental result. The interpretation via our method is only as good as the DL model used as input. Any similarities or discrepancies to the experimental data, represent what the DL model has learned about the AGO:miRNA:target interaction. Using our method, we can better evaluate the consistency of any DL model to this ground truth.

To compare the three interpretation methods, we computed the Pearson correlation coefficient between the experimental results and the importance scores calculated with each method based on the same DL model. Table 1 shows that results produced by our method positively correlate with the experimental results, while results computed by any of the in silico mutagenesis (ISM) methods correlate less positively, or even negatively.

### 3.3. Identifying Interaction Classes in CLASH Data

In the seminal CLASH paper [29] miRNA:target site interactions were clustered into interaction classes based on a per-nucleotide score derived from “cofold” analysis. Five classes with different binding profiles were produced, using k-means clustering (k = 5). Three of these classes (I–III) featured binding between the miRNA seed region and the target but differed in the presence and positioning of additional base-paired nucleotides within the miRNA. In class IV, binding was limited to a region located in the middle and 3′ end of the miRNA, denoting non-seed interactions. Class V showed distributed or less stable base pairing without either strong seed or 3′ binding.

**Figure 3:**
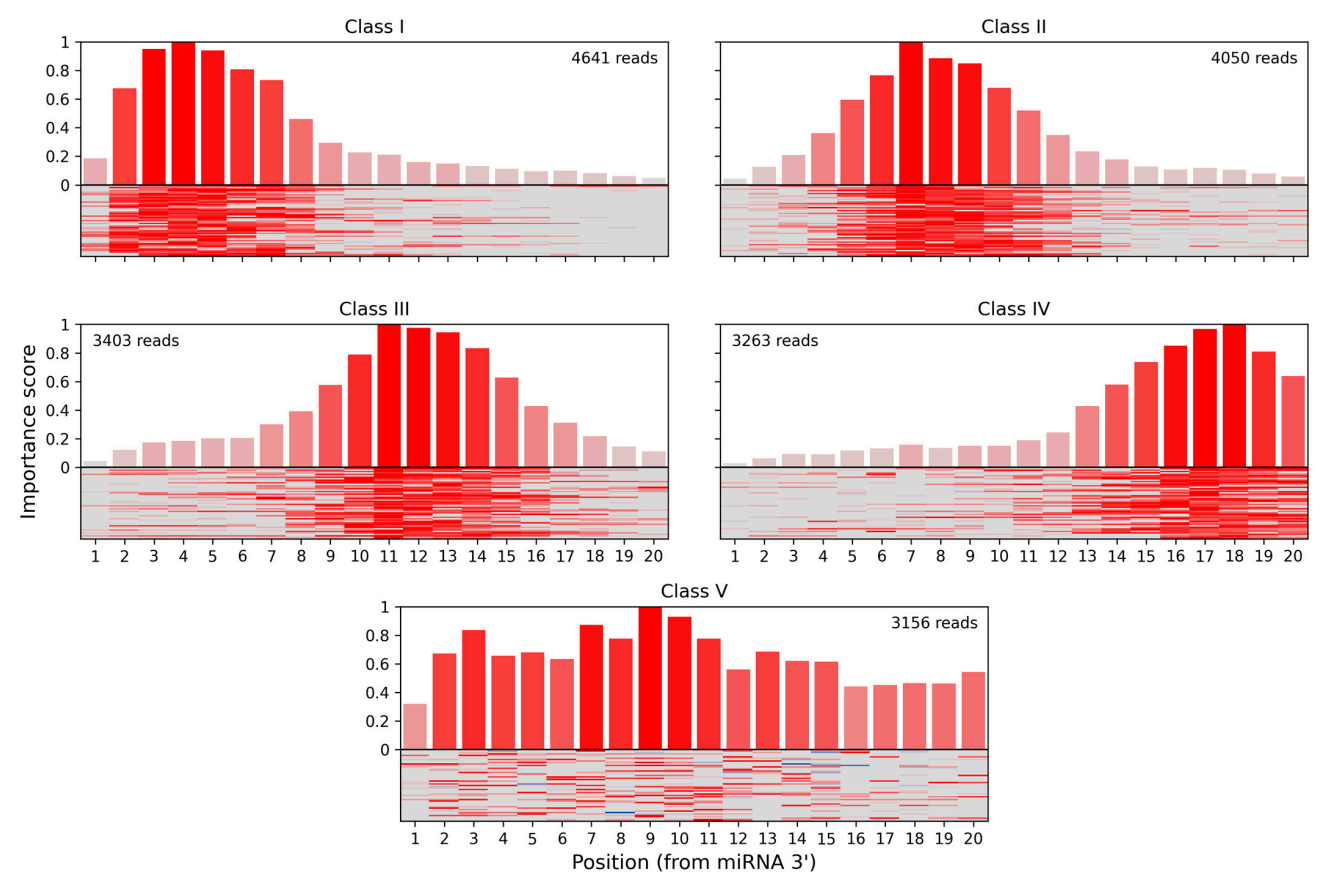
Classes of miRNA:binding site interactions with distinct base-pairing patterns computed for the Helwak et al., 2013 CLASH data using the miRBind model, DeepExplainer interpretation technique and our attribution sequence alignment method.

We have used the attribution scores produced by our method to reevaluate the rules of Ago1:miRNA:target binding learned by miRBind from the CLASH dataset. We calculated attribution scores for all CLASH interactions, based on the miRBind model, and then used k-means clustering (k = 5) to reveal five classes of interactions with distinct base-pairing patterns (Figure 3). Class I corresponded to the classical seed binding, while class II represented more relaxed seed binding. Classes III and IV showed binding in the middle and 3′ end of the miRNA, respectively, while class V showed a distributed base pairing pattern. CLASH interactions were almost uniformly distributed among classes, with 4641 in class I, 4050 in class II, 3403 in class III, 3263 in class IV, and 3156 in class V.

### 3.4. Attribution Scores Narrow down Binding Site Location Prediction

Target site prediction models such as miRBind are able to score miRNA:target site interactions of specific short lengths. However, the application of such methods on miRNA:target gene prediction is predicated on the ability to “scan” whole transcripts or other long RNA sequences. Our method can be used to make such “scanning” more precise, by narrowing down the binding site location.

**Figure 4:**
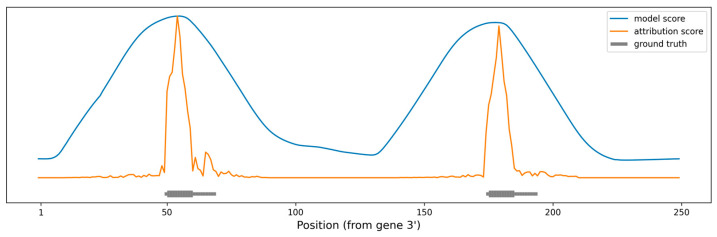
Scoring the positions in an artificial gene sequence to find areas with binding sites. The ground truth binding sites are shown in gray, with emphasis on the perfect 10 nt seed. The scoring obtained by scanning the gene with the miRBind model is shown in blue. The scoring obtained by scanning the gene with the miRBind model and interpreting it using the DeepExplainer are shown in orange.

As a proof of concept, we produced artificial RNA sequences of various lengths, with two perfect 10 nt miRNA seeds positioned at various distances between them. As a baseline, we used miRBind to “scan” the sequence using a moving window technique (see Methods for details). We also used our method to calculate attribution scores per nucleotide for the same sequences. Figure 4 shows the prediction made using each of the methods, along with the ground truth. The peaks produced by using miRBind scores are indeed covering the seed areas, but they are much wider than the actual binding sites. The peaks are not centered around the seeds and neither are the local maxima corresponding to the seed areas. In contrast, the peaks produced by using the attribution score point directly to, and are more tightly distributed around, the seed area.

**Figure 5:**
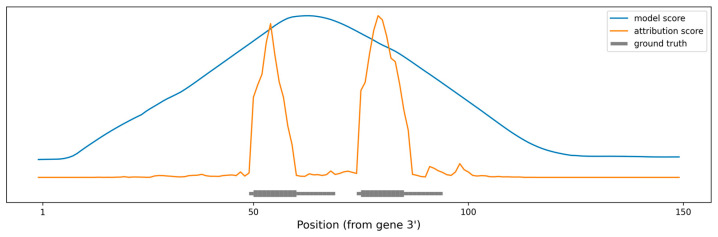
Scoring the positions in an artificial gene sequence to find areas with binding sites. The ground truth binding sites are shown in gray, with emphasis on the perfect 10 nt seed. The distance between starts of seeds is 25 nucleotides. The scoring obtained by scanning the gene with the miRBind model is shown in blue. The scoring obtained by scanning the gene with the miRBind model and interpreting it using the DeepExplainer are shown in orange.

Furthermore, the attribution score method can be even used to distinguish binding sites placed very closely together, for which miRBind model scores would produce only a single wide peak (Figure 5). We compared these two models on a dataset in which seeds were placed at the exact distances, from 15 nt to 50 nt apart. The attribution score model distinguishes the peaks even when the distance becomes as short as 15 nt (Figure S2).

**Figure 6:**
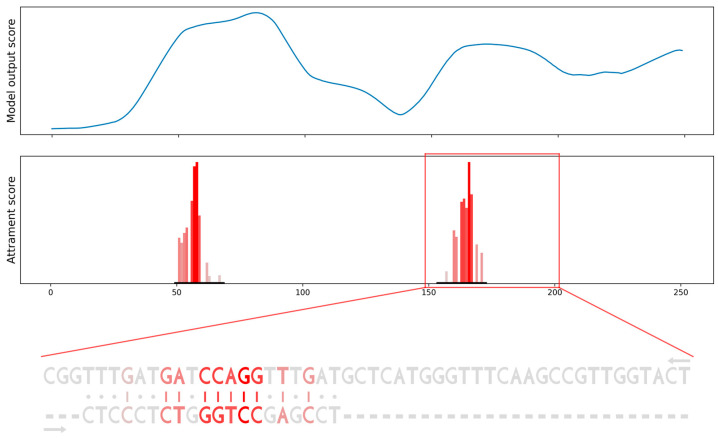
Scanning the gene for potential binding sites using the model’s output score compared to using our attribution sequence alignment. Model’s output scores (top row) point only. to the general area of binding sites. Attribution sequence alignment scores (bottom row) point to the specific binding sites, provide importance scores for each position in binding and visualize the interaction between miRNA and binding site as a sequence alignment.

To verify the results on more realistic data, we produced a sequence constructed from positive and negative binding sites of a specific miRNA derived from CLASH data. Again, the miRBind model’s output scores are able to roughly point to the positions of positive binding sites, but these peaks are wide, spanning more than 50 nt. When we computed the attribution score and the attribution sequence alignment, we were able to point to the exact position of miRNA binding. Moreover, we obtained the importance score for each position in the binding site and visualization of the interaction between miRNA and the binding site in the form of a sequence alignment (Figure 6).

### 3.5. Versatility of the Method

All previous results were produced using the miRBind trained model and the DeepExplainer interpretation method. However, our method is not tied to a specific model or interpretation method. To demonstrate this versatility, we used a different model (CNN_model_1_20_optimized) and a different interpretation method (GradientExplainer). We computed position importance scores for 300 miRNA:binding site pairs using different combinations of methods as inputs. DeepExplainer could not work with the CNN model, due to an implementation problem in its code. This highlights the importance of having a versatile method that can use different DL models, and interpretation methods. Table 2 shows that position importance scores computed by attribution sequence alignment method using different models and interpretation techniques positively correlate. A visual comparison of position importance scores for one sample is show in Supplementary Figure S3. Corresponding visualizations in the form of sequence alignments are shown in Supplementary Figure S4.

## 4. Discussion

Computational models, especially deep learning models, have become the state of the art in the classification of miRNA:target pairs. It is becoming increasingly important to be able to understand the reasoning behind their predictions. The use of a 2D-binding representation to encode interactions between two sequences has been a crucial innovation in miRNA:target prediction. Interpretation techniques can use this 2D-binding representation to produce maps of areas within the input that contribute positively or negatively to a model’s decision. However, it can be challenging to identify important biological features within this type of representation. The difficulty of interpretation of DL models compared to simpler co-fold or seed-based models may hold back their adoption as the state of the art in the miRNA target site prediction field, despite their superior predictive performance. For a DL model to be able to advance biological knowledge, a biologically relevant representation similar to sequence alignment is necessary. This type of representation is familiar to biologists, and easily human-readable, and can be used to condense the DL model’s focus into a small number of parameters. In this paper we introduce a novel interpretation technique called attribution sequence alignment which combines the principles of dynamic programming for semi-global sequence alignment with attribution scores obtained from interpreting a neural network trained on a 2D-binding representation. This method allows us to evaluate the importance of each individual nucleotide on a miRNA binding site, providing a biologically relevant representation that can be visualized as a sequence alignment.

Using this method, we can interpret DL models trained on miRNA:target site interaction. Our results correlate with in vivo experimental results and reveal interesting trends, such as the lower importance of 3’ nucleotides compared to the seed area and the low importance of the first nucleotide. However, it should be noted that these scores are specific to the model used and may vary with different models. Attribution sequence alignment scores can be a useful tool for understanding and evaluating the performance of a model, but they should not be considered a validation of the model itself. Further in vivo experimental results from systematically mutating miRNA target sites would be useful to calibrate interpretation methods such as ours more thoroughly.

The first step in any miRNA target prediction program is transcriptome-wide scanning for putative miRNA binding sites. These putative miRNA binding sites are further combined into a final prediction for each transcript. Using current miRNA:target site tools for transcriptome scanning are based on the DL giving a single score to a fixed size moving window (50 nt in the case of the miRBind model [28]) resulting in wide peaks. We demonstrate that attribution sequence alignment can be used for narrowing these peaks when scanning for binding sites by computing the miRNA:target site attribution sequence alignment and assigning per-nucleotide importance scores to a long sequence. Our method can provide target prediction programs with more specific and detailed information about each potential binding site, allowing it to leverage more information from the experimental data that has been encoded in the trained DL model.

The attribution sequence alignment method can be applied to the field of miRNA binding site prediction, as demonstrated by the miRBind model. However, it is not limited to this specific model, interpretation technique, or field. It could potentially be used for any neural network that has been trained on a 2D-binding representation of sequences, and any interpretation technique that produces per-pixel attribution scores. Additionally, with some modifications, it can easily be extended to other domains where input sequences can be represented by a 2D interaction matrix, such as protein–protein or protein–DNA interactions. Importantly, attribution sequence alignment considers only the scores from the interpretation matrix, without imposing any additional constraints on the alignment. This allows for greater flexibility and adaptability in its use.

## 5. Conclusions

In conclusion, we introduced a DL model interpretation method that can extract biologically relevant information from trained miRNA:target site prediction DL models. We demonstrated that this interpretation method can be used to interpret such models, as well as to narrow down their predictions on long target sequences. We believe that our method can facilitate the use of DL models for miRNA:target gene prediction, as well as the extraction of biological insight from DL models.

## Figures and Tables

**Table 1:** Comparison of experimentally obtained relative reporter activity values with values from three computational methods—our attribution sequence alignment, ISM Brennecke and ISM Full—using the Pearson correlation coefficient.

	Our	ISM Brennecke	ISM Full
mir-7 correlation	0.59	−0.09	−0.26
mir-278 correlation	0.85	NA	0.24

**Table 2:** Comparison of position importance scores computed by attribution sequence alignment using different models and interpretation techniques.

Methods	Mean	Standard Deviation	*p*-Value
miRBind DeepExplainer vs. miRBind GradientExplainer	0.86	0.23	1.51 × 10^−90^
miRBind DeepExplainer vs. CNN GradientExplainer	0.84	0.25	1.58 × 10^−88^
miRBind GradientExplainer vs. CNN GradientExplainer	0.79	0.26	1.96 × 10^−86^

## Data Availability

The data and code presented in this study are openly available at https://github.com/katarinagresova/DeepExperiment.

## Supplementary Materials

The following supporting information can be downloaded at: https://www.mdpi.com/article/10.3390/biology12030369/s1, Figure S1: Comparison of the effect of the number of background samples on computational time and variation in importance score; Table S1 Manually extracted values from Figure 1C from Brennecke et al., 2005; Figure S2: Scoring the positions in an artificial gene sequence to find areas with binding sites; Figure S3: Visual comparison of position importance scores computed for one sample using the miRBind model or CNN model, and using the GradientExplainer or DeepExplainer interpretation technique; Figure S4: Interaction between miRNA and binding site visualize as sequence alignment.
