# Supplementary material for: Using Attribution Sequence Alignment to Interpret Deep Learning Models for miRNA Binding Site Prediction
Source: Biology (Basel). 2023 Feb 26;12(3):369. doi: 10.3390/biology12030369 (PMC10045089; doi:10.3390/biology12030369)
Supplement: Supplementary file 1 [file biology-12-00369-s001.zip › TableS1.pdf]

Table S1. Manually extracted values from Figure 1C from Brennecke et al., 2005. The “mean” columns contain the values of bars, the “low” columns values of the low ends of error bars, and the “high” columns values of the high ends of error bars.

Mismatch position	miR7 low	miR7 mean	miR7 high	miR278 low	miR278 mean	miR278 high
0	8.4	12.5	16.6	9.3	10	10.5
1	6.8	9.8	12.5	8.1	13.4	19
2	77.5	80	82.8	25.9	36.1	46.7
3	90.1	93.4	95.9	37.8	52.8	67.4
4	64.9	68.7	71.9	51.9	56.6	61.3
5	66.2	68.2	69.8	33.6	47.4	60.8
6	85.3	88.5	91.4	66.2	73.5	81.2
7	70.3	74.2	78.8	24.7	51.2	77.2
8	57.6	64.2	71.1	10.5	15.7	21.1
9	15.4	16.5	17.8	5.5	13.4	21.1
10	29.5	31.7	34	0	0	0
3'	29.5	32.4	34.9	21.1	26.7	32.4
