# Supplementary figures and images for: Using Attribution Sequence Alignment to Interpret Deep Learning Models for miRNA Binding Site Prediction
Source: Biology (Basel). 2023 Feb 26;12(3):369. doi: 10.3390/biology12030369 (PMC10045089; doi:10.3390/biology12030369)

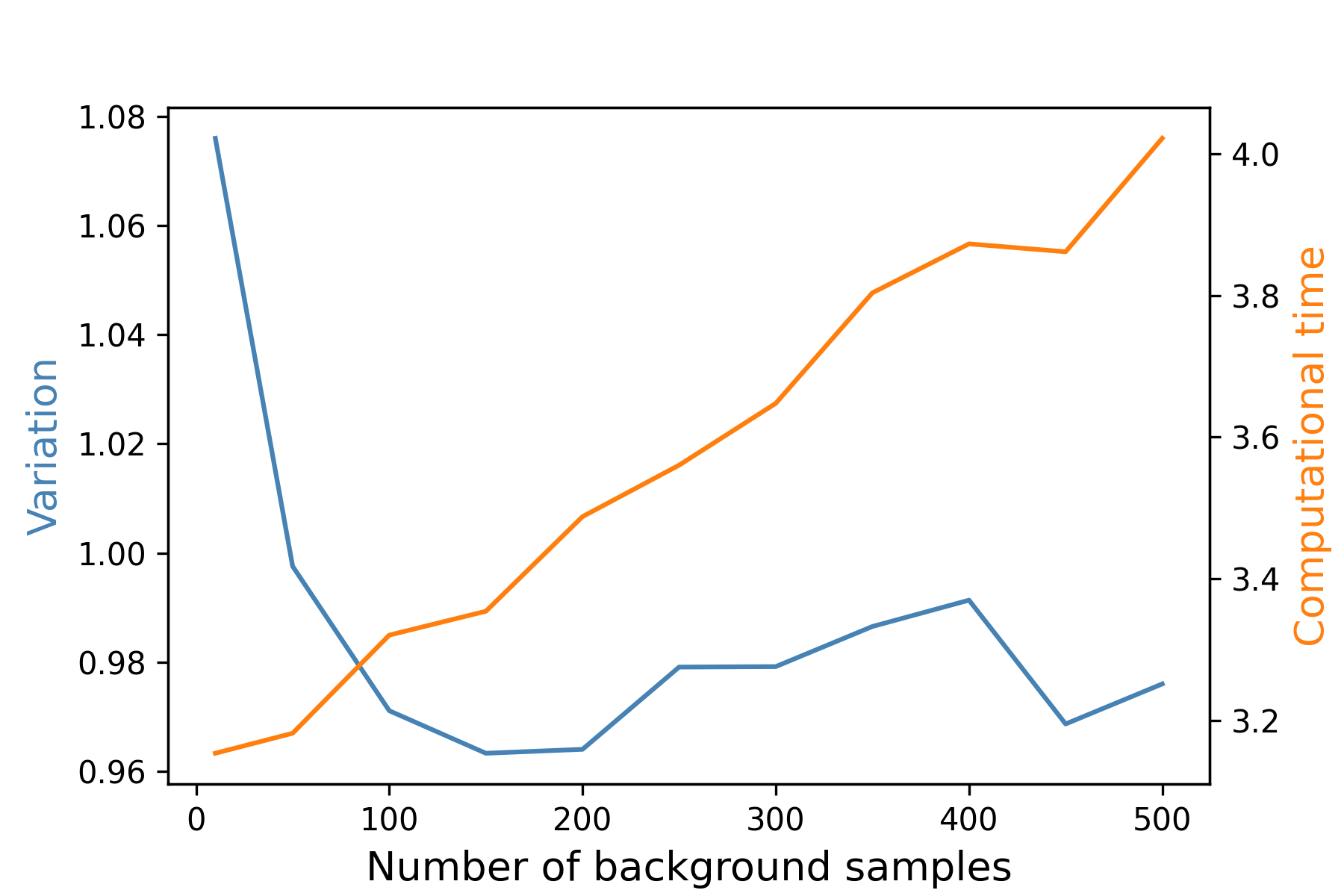

Supplement: Supplementary file 1 [file biology-12-00369-s001.zip › FigureS1_variation.png]

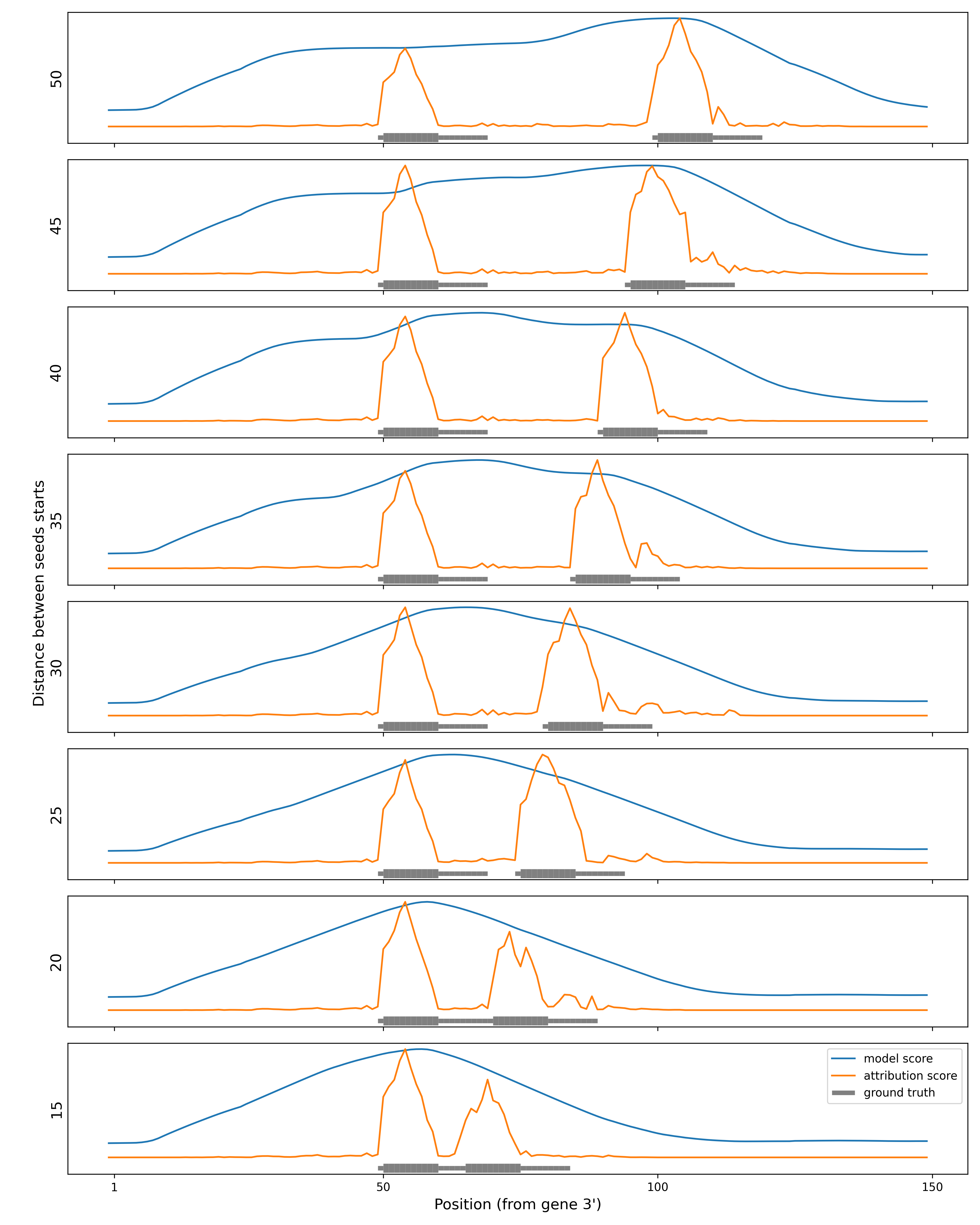

Supplement: Supplementary file 1 [file biology-12-00369-s001.zip › FigureS2_closeness_analysis.png]

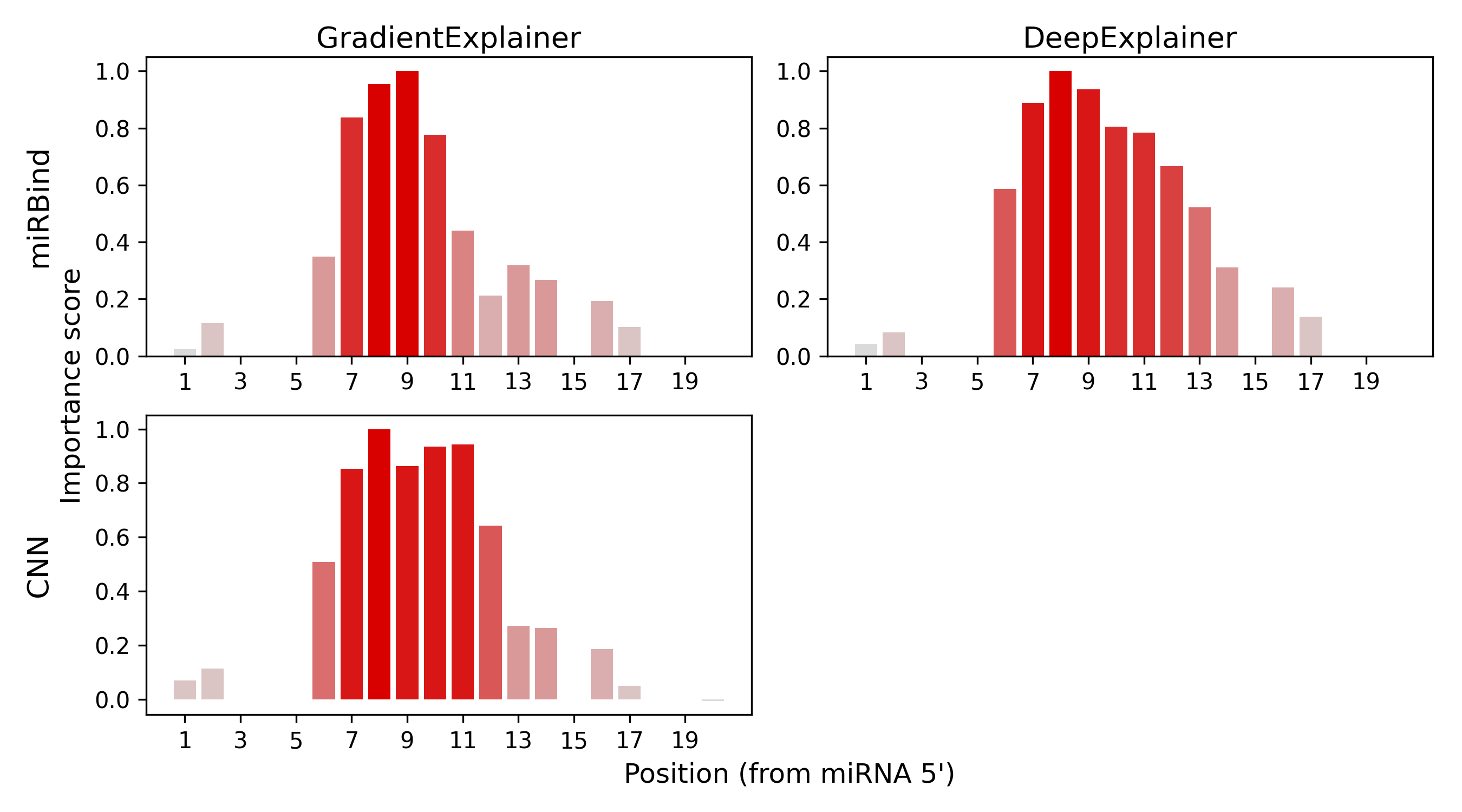

Supplement: Supplementary file 1 [file biology-12-00369-s001.zip › FigureS3_versatility.png]

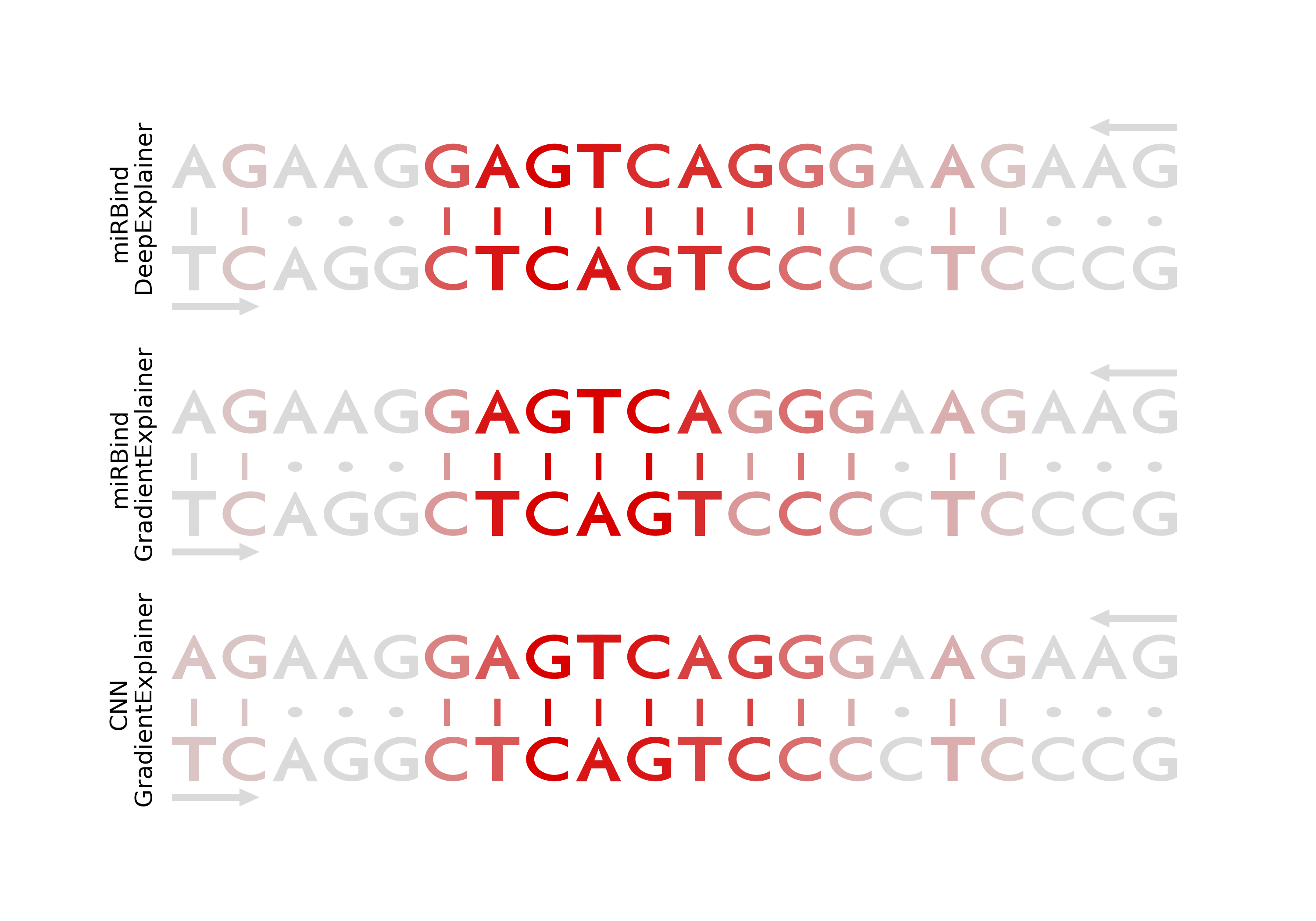

Supplement: Supplementary file 1 [file biology-12-00369-s001.zip › FigureS4_alignment_comparison.png]
